# Disrupted basal forebrain-cortical connectivity in amnestic mild cognitive impairment: unveiling circuit-level links to cognitive decline

**DOI:** 10.3389/fneur.2026.1846663

**Published:** 2026-06-16

**Authors:** Rong-Rong Huang, Jie Ma, Jia-Jia Wu, Mou-Xiong Zheng, Juan-Juan Lu, Jing Jin, Xu-Yun Hua, Jian-Guang Xu

**Affiliations:** 1 Department of Rehabilitation Medicine, Yueyang Hospital of Integrated Traditional Chinese and Western Medicine, Shanghai University of Traditional Chinese Medicine, Shanghai, China; 2 Department of Traumatology and Orthopedics, Shuguang Hospital Affiliated to Shanghai University of Traditional Chinese Medicine, Shanghai, China; 3 School of Rehabilitation Science, Shanghai University of Traditional Chinese Medicine, Shanghai, China

**Keywords:** amnestic mild cognitive impairment, basal forebrain, fMRI, functional connectivity, neural circuits

## Abstract

**Background:**

Amnestic Mild Cognitive Impairment (aMCI) is a transitional stage between normal individuals and dementia. The basal forebrain is a key subcortical hub involved in memory-related cortico-subcortical networks.

**Methods:**

A total of 66 aMCI patients, including 5 single-domain and 61 multidomain aMCI patients, and 59 healthy controls (HCs) were recruited from the community and underwent comprehensive neuropsychological assessments. After excluding one HC participant due to excessive head motion, 66 aMCI patients and 58 HCs were included in the final imaging analysis. Resting-state functional magnetic resonance imaging (rs-fMRI) was performed, and seed-based functional connectivity analysis was conducted with the basal forebrain as the region of interest.

**Results:**

Compared with HCs, aMCI patients exhibited reduced basal forebrain functional connectivity overall, but showed increased connectivity between the right nucleus basalis of Meynert and specific cortical regions (*p* < 0.01, GRF corrected). Long-delayed recall was associated with connectivity between the basal forebrain and the left postcentral gyrus, while language and executive functions were associated with connectivity in the left inferior frontal gyrus (opercular part) and the calcarine cortex, respectively (*p* < 0.05).

**Conclusion:**

These findings may help identify basal forebrain-associated network alterations as potential targets for future circuit-based neuromodulation studies in aMCI.

## 1 Introduction

With the acceleration of global population aging, cognitive impairment has become a major factor affecting the quality of life in older adults. Its severity is closely associated with aging, leading not only to higher risks of hospitalization and mortality but also imposing a substantial socio-economic burden (1). Mild cognitive impairment (MCI) is recognized as a transitional stage between normal aging and dementia. Although individuals with MCI can generally maintain basic independence in daily activities, they are at markedly increased risk of progressing to dementia, particularly those with the amnestic mild cognitive impairment (aMCI) (2, 3). Importantly, patients with aMCI are considered ideal candidates for both pharmacological and non-pharmacological interventions, making this subgroup a key target for research (2).

Episodic memory impairment, especially deficits in long-delayed recall, has been consistently identified as the core cognitive feature of aMCI (4). Episodic memory is essential for recalling the spatiotemporal context of past experiences (5). Recent studies have revealed that memory function is supported by interconnected neural circuits rather than localized to a single brain region. Meta-analyses have shown abnormal spontaneous activity in multiple memory-related brain regions in aMCI (6). At the network level, structural and functional alterations have been observed in the dorsal attention, default mode, salience, and executive control networks (7, 8). This evidence reflects a shift in contemporary memory research from a regional to a circuit-level perspective. Notably, circuit-based neuromodulation studies have demonstrated that 2 weeks of transcranial magnetic stimulation (TMS) can significantly improve long-delayed recall in aMCI patients (9). These findings highlight potential therapeutic targets such as the hippocampus and precuneus. However, given the complexity of memory-related synaptic mechanisms, further exploration of additional neural pathways is crucial to advance our understanding and identify novel targets for intervention.

Among the neural circuits implicated in cognitive decline, the basal forebrain has received increasing attention due to its widespread projections to the cerebral cortex, hippocampus, and amygdala (10). The basal forebrain is a subcortical structure located beneath the anterior commissure and includes several key nuclei: the substantia innominata, nucleus accumbens, ventral pallidum, bed nucleus of the stria terminalis, preoptic area, nucleus basalis of Meynert (NBM), diagonal band of Broca (DB), and septal nuclei (11). It contains multiple neuronal populations and participates in large-scale cortico-subcortical circuits through both efferent and afferent pathways (12). Therefore, alterations in basal forebrain connectivity may provide systems-level information about circuit dysfunction related to attention, learning, memory, arousal, and perceptual processing.

A major challenge is that the basal forebrain lies deep within the brain and lacks clear anatomical boundaries, complicating its delineation with conventional MRI (13). However, in 2008, Zaborszky et al. proposed a stereotaxic probabilistic map of the basal forebrain region, based on postmortem anatomical studies of magnocellular cell groups (14). They identified these cell groups on histological sections from 10 postmortem human brains and reconstructed them in three dimensions. Further anatomical studies have subdivided basal forebrain into four groups (Ch1–Ch4) in primates (15). Specifically, Ch1 (medial septum) and Ch2 (vertical limb of DB) project to the hippocampus, Ch3 (horizontal limb of DB) projects mainly to the olfactory bulb, and Ch4 (NBM) provides extensive projections to the association cortex and amygdala (16). This topographic organization provides a structural basis for investigating the functional roles of distinct basal forebrain pathways in memory and cognition.

Our research aims to use resting-state functional magnetic resonance imaging (rs-fMRI) to explore basal forebrain functional connectivity in aMCI patients. By characterizing alterations in basal forebrain-centered circuits, our work aims to elucidate the neural mechanisms underlying episodic memory impairment and identify potential targets for early intervention in aMCI patients.

## 2 Materials and methods

This case–control study was approved by the Institutional Ethics Committee of Yueyang Hospital of Integrated Traditional Chinese and Western Medicine, Shanghai University of Traditional Chinese Medicine (Approval No.: 2021-103). A total of 125 participants were recruited from surrounding communities between 2021 and 2022. Written informed consent was obtained from all participants before study enrollment. No patients or members of the public were involved in the design, conduct, reporting or dissemination of this study.

### 2.1 Inclusion and exclusion criteria

Inclusion criteria for the aMCI group were as follows: (1) age between 50 and 80 years; (2) diagnosis of aMCI according to the Petersen/Winblad criteria (17, 18). (3) Self-reported subjective cognitive complaints; (4) first diagnosed with aMCI and no prior use of cognitive-enhancing medications; (5) a Clinical Dementia Rating (CDR) memory score of 0.5, not meeting the dementia diagnostic criteria of the National Institute on Aging and Alzheimer’s Association (NIA-AA). Inclusion criteria for the healthy controls (HCs) included: (1) age between 50 and 80 years; (2) No subjective cognitive complaints; (3) all neuropsychological test results within the normal range.

Exclusion criteria for both groups were: (1) neurological disorders such as stroke or Parkinson’s disease; (2) history of psychiatric disease; (3) current or prior use of cognitive-enhancing medications; (4) severe cardiovascular, renal, hepatic, or oncological disease; (5) inability to complete neuropsychological assessments; (6) contraindications to MRI scanning (e.g., pacemaker, metallic stents, or dental implants).

### 2.2 Neuropsychological assessment

Assessments were conducted by two senior neuropsychologists (each with >10 years of clinical experience) blinded to participants’ clinical diagnoses. A third neuropsychologist independently reviewed the results. Each participant completed a comprehensive evaluation, including: Mini-Mental State Examination (MMSE) for global cognition (19); Auditory Verbal Learning Test (AVLT) for episodic memory (AVLT) cumulative score as total recall; AVLT_N5 score for long-delayed recall (20); Symbol Digit Modalities Test (SDMT) for attention; Shape Trail Test Part B (STT-B) for information processing speed and executive function (21); Boston Naming Test (BNT) for visual–verbal ability (22); Hamilton Depression Rating Scale (HAMD) (23) and Hamilton Anxiety Rating Scale (HAMA) (24) for emotional status.

### 2.3 Data acquisition

Brain imaging data were acquired using a MAGNETOM Verio 3 T MRI scanner (Siemens Healthcare, Erlangen, Germany). Resting-state functional MRI (rs-fMRI) images were obtained using a gradient-echo echo-planar imaging (GRE-EPI) sequence with the following parameters: repetition time (TR) = 3,000 ms; echo time (TE) = 30 ms; flip angle = 90°; matrix size = 64 × 64; field of view (FOV) = 230 mm × 230 mm; slice thickness = 3.0 mm; voxel size = 3.6 mm × 3.6 mm × 3.0 mm; number of slices = 43; slice acquisition = interleaved; number of volumes = 240.

### 2.4 Data preprocessing

Data preprocessing was performed using RESTplus software on the MATLAB 2021a platform. The steps included: (1) data format conversion: converting data to NIfTI format using dcm2nii; (2) quality check: removing the first 10 time points to mitigate effects from scanner warm-up or head motion. (3) Slice timing correction: applying interleaved ascending odd slice order correction. (4) Realignment: correcting for head motion to reduce spatial displacement. Participants were excluded if maximum head motion exceeded 3 mm translation or 3° rotation. (5) Spatial normalization: Normalizing images to standard MNI space. (6) Nuisance covariate regression: regressing out nuisance signals, including head motion parameters, white matter signals, and cerebrospinal fluid signals. (7) Temporal filtering: applying band-pass filtering at 0.01–0.08 Hz. (8) Smoothing: a Gaussian kernel with a full-width at half-maximum (FWHM) of 6 mm was applied to smooth the functional images and improve the signal-to-noise ratio.

### 2.5 Seed-based functional connectivity analysis

**Figure 1:**
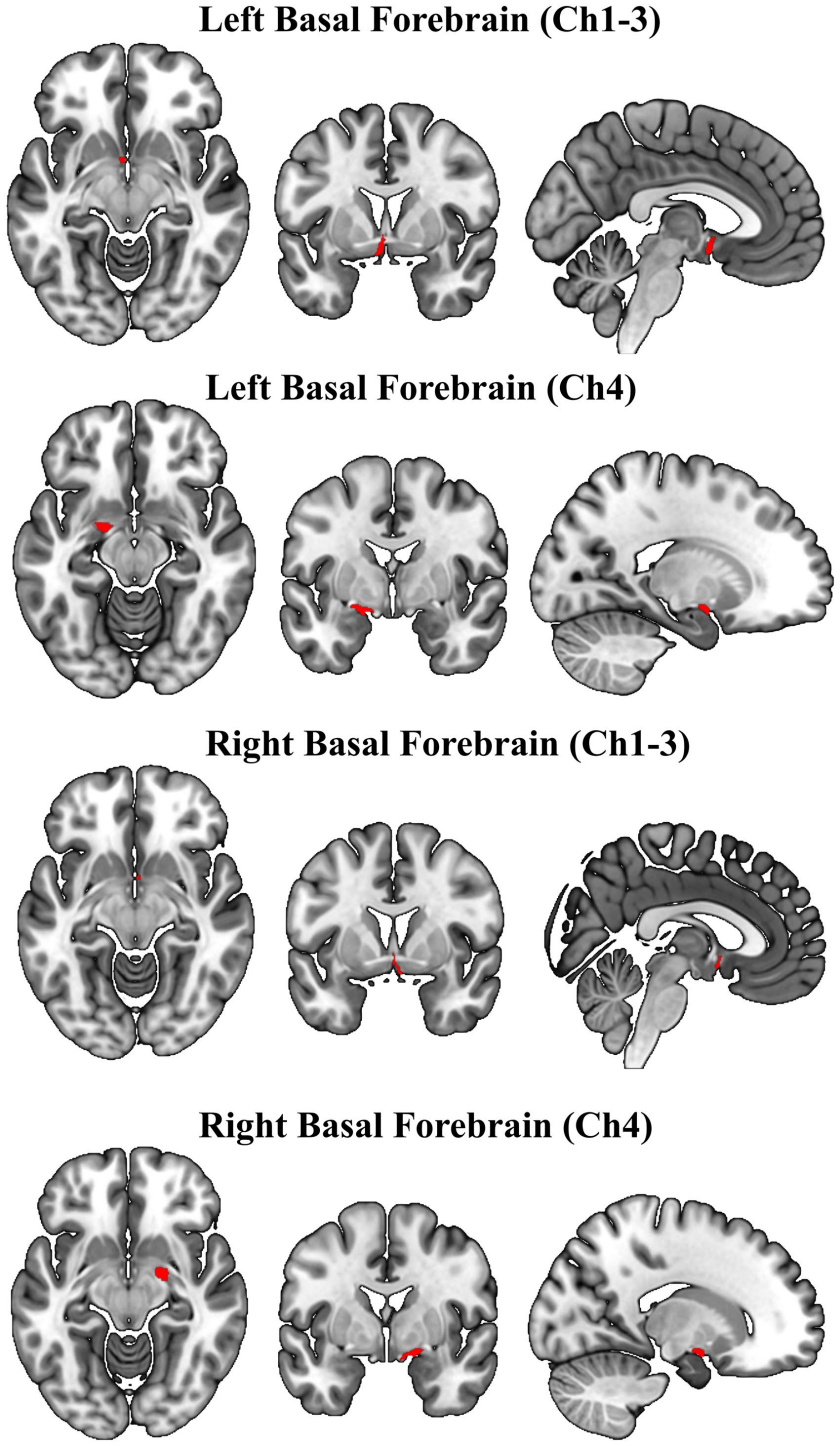
Anatomical diagram of basal forebrain.

The probabilistic map of the basal forebrain was extracted from the SPM Anatomy Toolbox^1^ based on Zaborszky et al. (14). Four regions of interest (ROIs) were defined: Left Basal Forebrain (Ch1-3) (Ch1-3: medial septum and diagonal band), Left Basal Forebrain (Ch4) (Ch4: NBM), Right Basal Forebrain (Ch1-3), and Right Basal Forebrain (Ch4) (Figure 1). These ROIs were registered to MNI space and used as seed masks.

Seed-wise functional connectivity (FC) analysis was performed with RESTplus using the Anatomical Automatic Labeling (AAL90) atlas. For each ROI, the mean time series was extracted and correlated with whole-brain voxels. Correlation coefficients were Fisher’s z-transformed, and resulting zFC maps were smoothed with a 4 mm FWHM Gaussian kernel.

### 2.6 Statistical analysis

Statistical analyses of demographic and neuropsychological data were performed using SPSS 27. Demographic variables (age, gender, education, height, weight) were tested for normality. Normally distributed variables were expressed as mean ± standard deviation (SD); non-normally distributed data were analyzed with non-parametric tests. Categorical variables (e.g., smoking, alcohol consumption, hypertension, hyperlipidemia, type 2 diabetes) were compared with chi-square tests. Cognitive and emotional scale scores (MMSE, AVLT, AVLT_N5, SDMT, STT-B, BNT, HAMD, HAMA) were compared between groups using two-tailed tests (*p* < 0.05).

Voxel-wise functional connectivity (FC) statistical analysis was performed using SPM 12 on the MATLAB 2021a platform. At the second level, a two-sample *t*-test was employed to compare the two groups, with years of education entered as a covariate in the general linear model (25–27). Multiple comparisons were corrected using Gaussian Random Field (GRF) theory, with a two-tailed voxel-level threshold of *p* < 0.01 and cluster-level correction at *p* < 0.01. Additionally, the extracted time series were correlated with neuropsychological scores (*p* < 0.05).

## 3 Results

### 3.1 Statistical analysis of baseline data

Participants were initially classified into two groups: 66 aMCI patients and 59 HCs. One HC participant was excluded from imaging analyses due to excessive head motion, leaving 66 aMCI patients, including 5 single-domain and 61 multidomain aMCI patients, and 58 HCs for the final analysis. Baseline demographic and clinical variables were compared between the final analysis groups. Significant group differences were found in years of education and neuropsychological measures, including MMSE, AVLT, AVLT_N5, SDMT, STT-B, BNT, and HAMD (Table 1). No significant differences were observed in age, height, weight, smoking, drinking, hypertension, hyperlipidemia, or type 2 diabetes.

**Table 1:** Comparison of clinical characteristics between aMCI and HC groups.

Characteristic	aMCI (*n* = 66)	HC (*n* = 58)	*T*/*Z*/*χ*^2^	*p*-value
Basic characteristics				
Age, median (Q1, Q3)	66.5 (62, 71)	66.5 (60.75, 71)	0.569	0.569
Gender, *n* (%)	36 (54.54%)	29 (50.87%)	0.234	0.629
Education, median (Q1, Q3)	9 (9,12)	11.5 (9,14)	2.313	0.021
Height, median (Q1, Q3)	162.5 (155.75, 170)	165.5 (157.75, 170)	1.031	0.303
Weight, mean ± SD	64.65 ± 10.66	64.34 ± 9.21	0.171	0.864
Smoking, *n* (%)	16 (24.24%)	14 (24.14%)	−0.43	0.836
Drinking, *n* (%)	7 (10.6%)	11 (18.97%)	−1.738	0.187
Hypertension, *n* (%)	28 (42.42%)	26 (44.82%)	−0.073	0.788
Hyperlipidemia, *n* (%)	47 (71.21%)	42 (72.41%)	−0.220	0.882
Type 2 diabetes, *n* (%)	49 (74.42%)	47 (81.03%)	−0.815	0.367
General cognitive function				
MMSE, median (Q1, Q3)	26 (24, 28)	27 (25, 29)	4.514	<0.001
Memory function				
AVLT, mean ± SD	16.53 ± 4.89	31.23 ± 7.38	−13.224	<0.001
AVLT_N5, median (Q1, Q3)	2 (1, 3)	6 (5, 7)	9.282	<0.001
Attention function				
SDMT, median (Q1, Q3)	27 (19.75, 32.25)	39 (33.75, 44)	6.908	<0.001
Executive function				
STT-B, median (Q1, Q3)	164.95 (124.25, 245.50)	115.50 (99.14, 135.07)	4.81	<0.001
Language function				
BNT, median (Q1, Q3)	21 (18, 25)	25 (23, 26)	4.74	<0.001
Emotional performance				
HAMD, median (Q1, Q3)	1 (0, 3)	0 (0, 2)	2.001	0.045
HAMA, median (Q1, Q3)	3 (1, 5)	2 (1, 4)	1.380	0.168

aMCI, amnestic mild cognitive impairment; HC, healthy control; MMSE, Mini-Mental State Examination; AVLT, Auditory Verbal Learning Test Total Recall (sum of AVLT_N1, AVLT_N2, AVLT_N3, AVLT_N4 and AVLT_N5); AVLT_N5, Auditory Verbal Learning Test Fifth Trial (long-delayed recall); SDMT, Symbol Digit Modalities Test; STT-B, Shape Trail Test, part B; BNT, Boston Naming Test; HAMD, Hamilton Depression Scale; HAMA, Hamilton Anxiety Scale.

### 3.2 Functional connectivity of left basal forebrain (Ch1-3)

**Figure 2:**
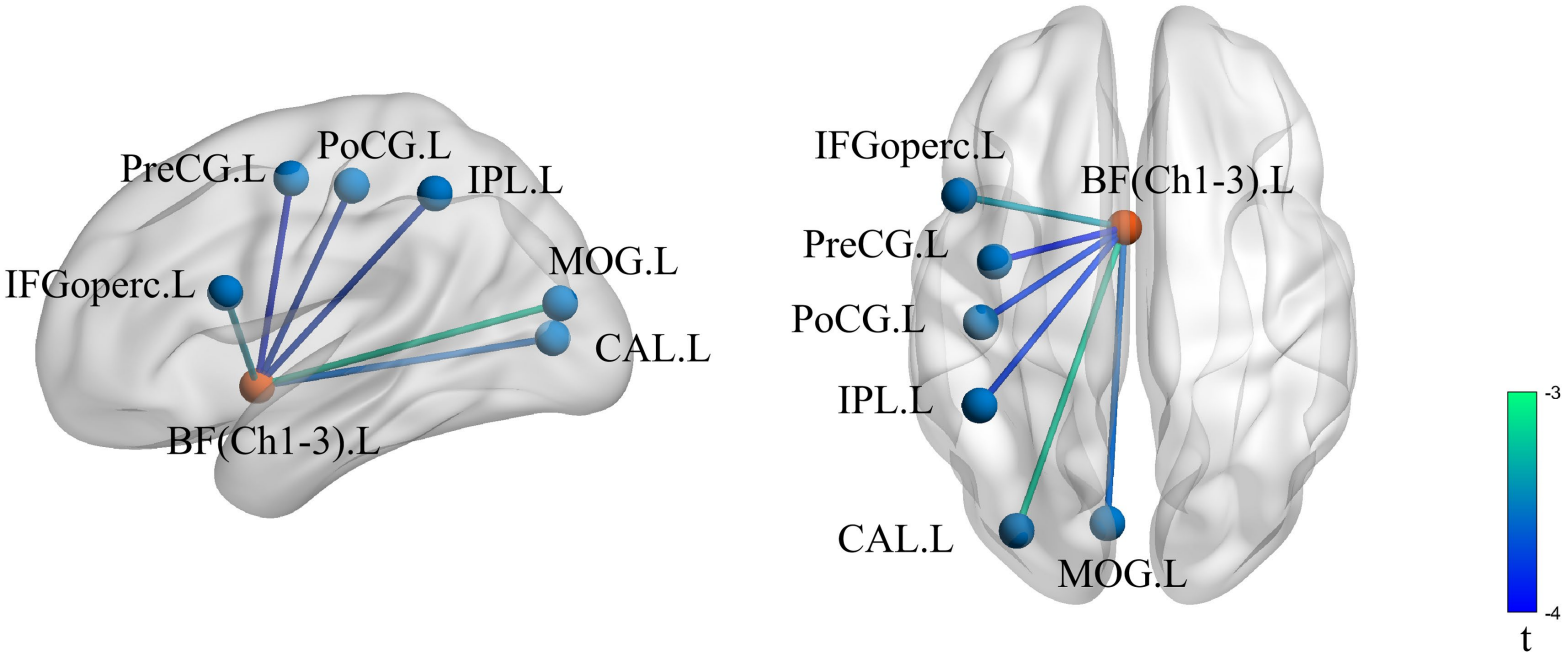
Whole-brain functional connectivity of left Basal Forebrain (Ch1-3) in the aMCI patients compared with the HCs. The blue color of edge indicates decreased connectivity voxel-level *p* < 0.01, cluster-level *p* < 0.01, GRF corrected. Regions include the left postcentral gyrus, left precentral gyrus, opercular part of the left inferior frontal gyrus, left inferior parietal lobule, left calcarine cortex, and left middle occipital gyrus. PoCG_L, Left postcentral gyrus; PreCG_L, Left precentral gyrus; IFGoperc_L, Opercular part of the left inferior frontal gyrus; IPL_L, Left inferior parietal lobule; CAL_L, Left calcarine; MOG_L, Left middle occipital gyrus; BF (Ch1-3). L, Left Basal Forebrain (Ch1-3).

Compared with HCs, the aMCI group showed significantly reduced functional connectivity between left Basal Forebrain (Ch1-3) and the left postcentral gyrus, precentral gyrus, opercular part of the inferior frontal gyrus, inferior parietal lobule, calcarine cortex, and middle occipital gyrus (voxel-level *p* < 0.01, cluster-level *p* < 0.01, GRF corrected) (Figure 2).

### 3.3 Functional connectivity of left basal forebrain (Ch4)

**Figure 3:**
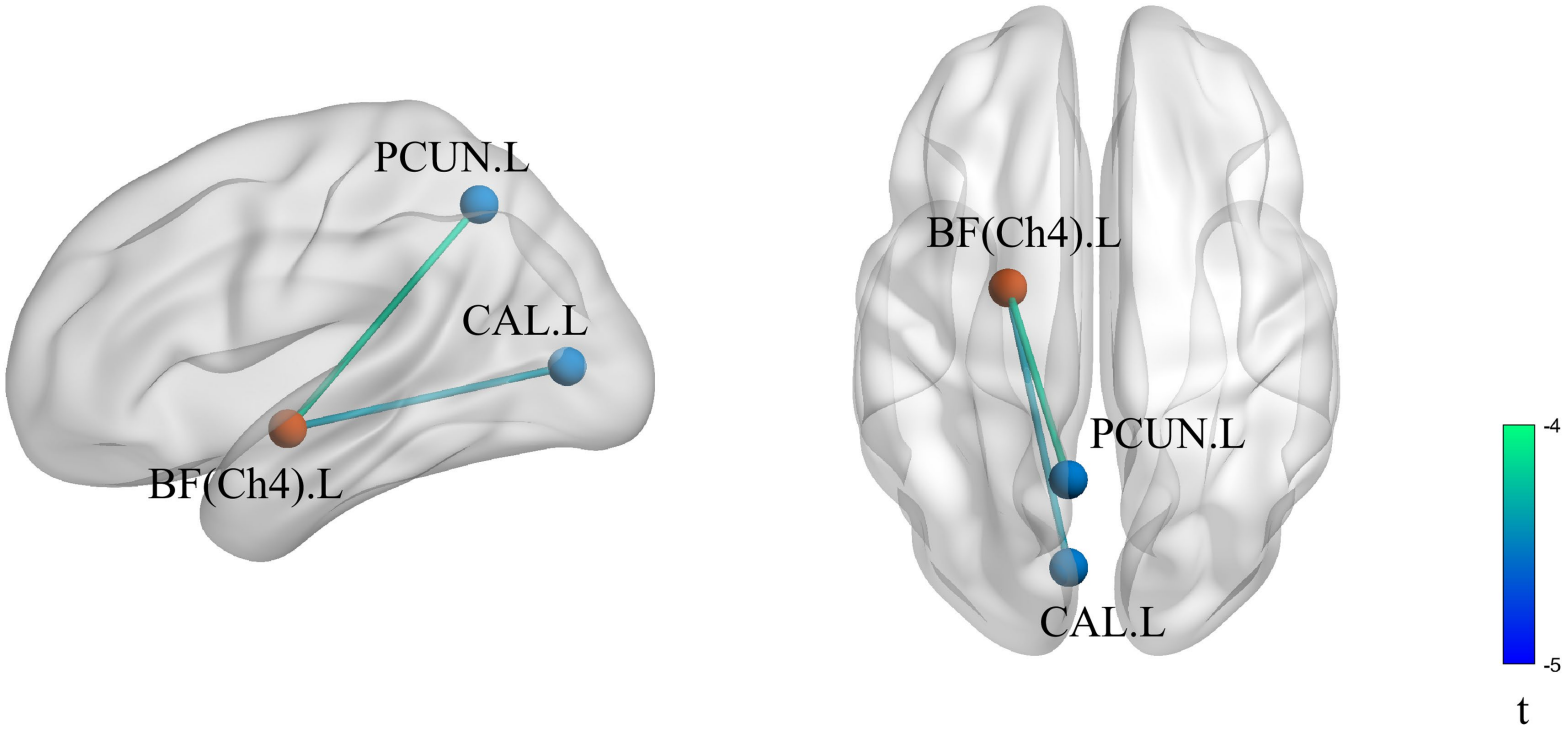
Whole-brain functional connectivity of left Basal Forebrain (Ch4) in the aMCI patients compared with the HCs. The blue color of edge indicates decreased connectivity (voxel-level *p* < 0.01, cluster-level *p* < 0.01, GRF corrected). Significant clusters included the left calcarine cortex and the left precuneus. CAL_L, Left calcarine; PCUN_L, Left precuneus; BF (Ch4). L, Left Basal Forebrain (Ch4).

Functional connectivity between left Basal Forebrain (Ch4) and the left calcarine cortex and precuneus was significantly reduced in aMCI patients (voxel-level *p* < 0.01, cluster-level *p* < 0.01, GRF corrected) (Figure 3).

### 3.4 Functional connectivity of right basal forebrain (Ch1-3)

**Figure 4:**
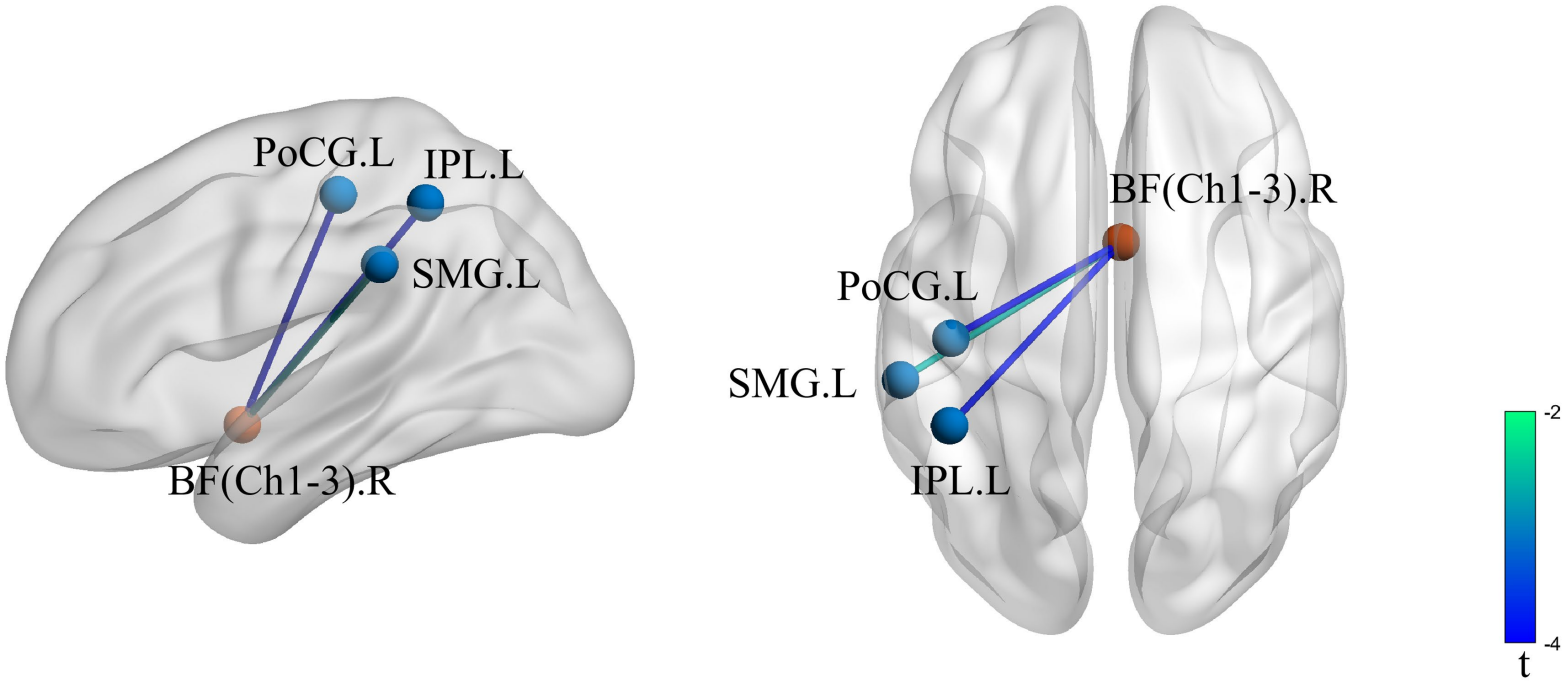
Whole-brain functional connectivity of right Basal Forebrain (Ch1-3) in the aMCI patients compared with the HCs. The blue color of edge indicates decreased connectivity (voxel-level *p* < 0.01, cluster-level *p* < 0.01, GRF corrected). Significant clusters were identified in the left postcentral gyrus, left supramarginal gyrus, and left inferior parietal lobule. PoCG_L, Left postcentral gyrus; SMG_L, Left supramarginal gyrus; IPL_L, Left inferior parietal lobule; BF (Ch1-3). R, Right Basal Forebrain (Ch1-3).

The aMCI group showed decreased connectivity between right Basal Forebrain (Ch1-3) and the left postcentral gyrus, supramarginal gyrus, and inferior parietal lobule (voxel-level *p* < 0.01, cluster-level *p* < 0.01, GRF corrected) (Figure 4).

### 3.5 Functional connectivity of right basal forebrain (Ch4)

**Figure 5:**
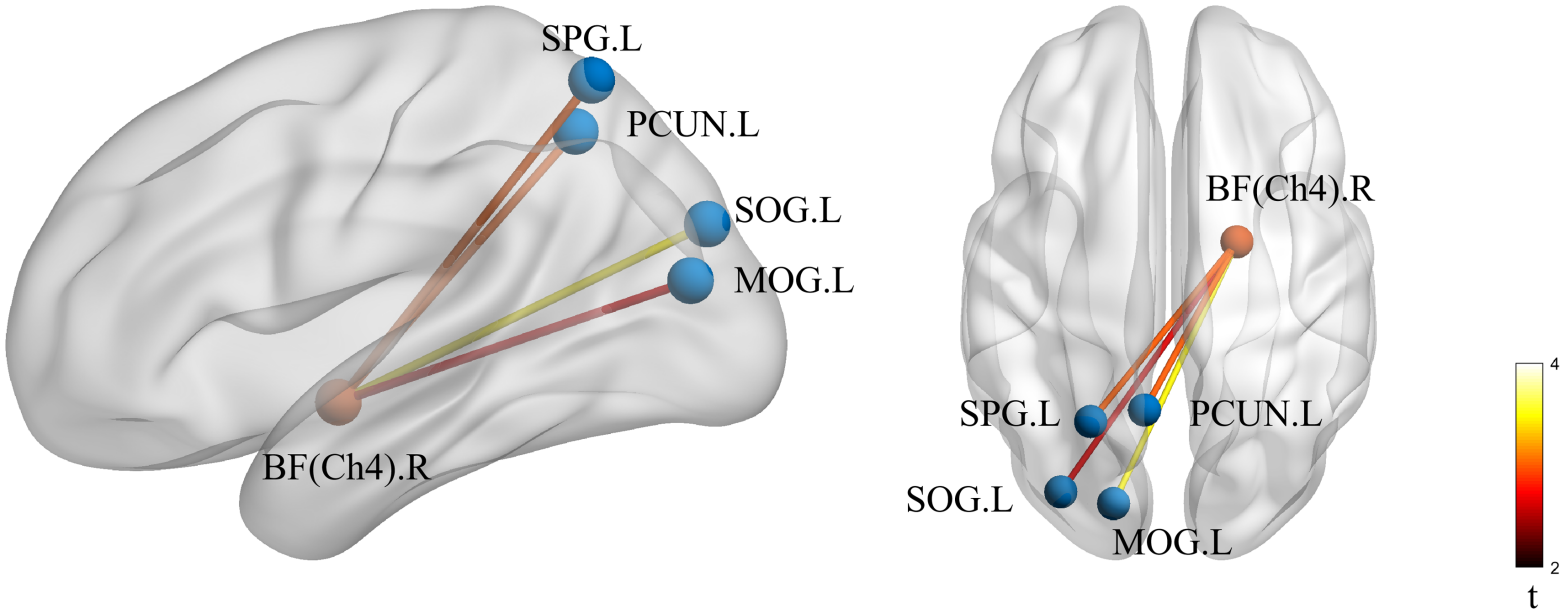
Whole-brain functional connectivity of right Basal Forebrain (Ch4) in the aMCI patients compared with the HCs. The red color of edge indicates increased connectivity (voxel-level *p* < 0.01, cluster-level *p* < 0.01, GRF corrected). Significant clusters included the left superior occipital gyrus, left middle occipital gyrus, left precuneus, and left superior parietal lobule. SOG_L, Left superior occipital gyrus; MOG_L, Left middle occipital gyrus; PCUN_L, Left precuneus; SPG_L, Left superior parietal lobule; BF (Ch4). R, Right Basal Forebrain (Ch4).

In contrast, functional connectivity between right Basal Forebrain (Ch4) and the left superior occipital gyrus, middle occipital gyrus, precuneus, and superior parietal lobule was significantly increased in aMCI patients compared with HCs (voxel-level *p* < 0.01, cluster-level *p* < 0.01, GRF corrected) (Figure 5).

### 3.6 Correlation analysis

**Figure 6:**
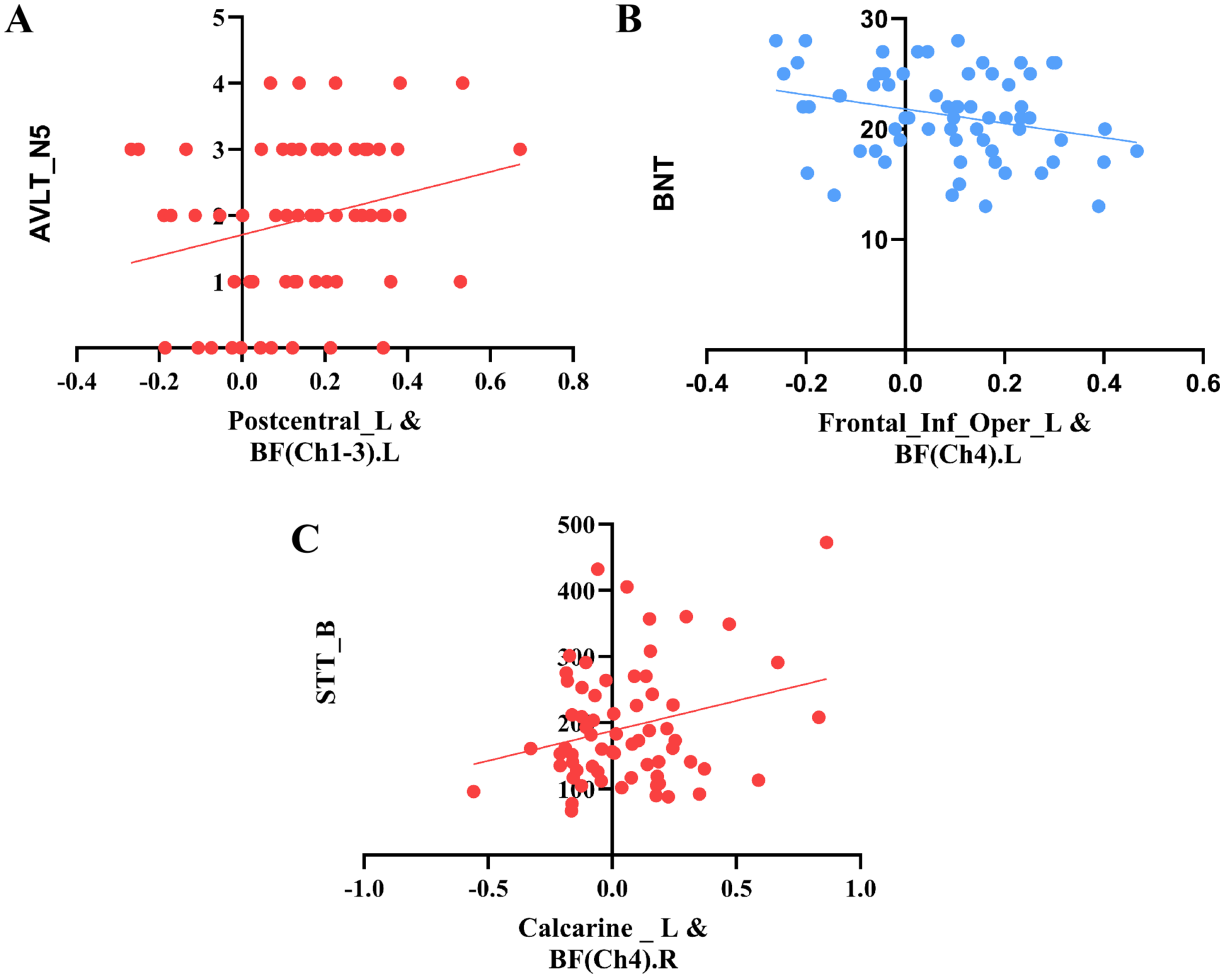
Correlation analysis between basal forebrain connectivity and neuropsychological performance. Red indicates positive correlation; blue indicates negative correlation. Associations were observed between (1) Left Basal Forebrain (Ch1-3)—Left postcentral gyrus connectivity and AVLT_N5 scores, (2) Left Basal Forebrain (Ch4)—Left inferior frontal gyrus (opercular part) connectivity and BNT scores, and (3) Right Basal Forebrain (Ch4)—Left calcarine cortex connectivity and STT-B scores. Postcentral_L, Left postcentral gyrus; Frontal_Inf_Oper_L, Opercular part of the left inferior frontal gyrus; Calcarine_L, Left calcarine; BF (Ch1-3). L, Left Basal Forebrain (Ch1-3); BF (Ch4). L, Left Basal Forebrain (Ch4); BF (Ch4). R, Right Basal Forebrain (Ch4); AVLT_N5, Auditory Verbal Learning Test (long-delayed recall); BNT, Boston Naming Test; STT-B, Shape Trail Test Part B.

Correlation analysis results showed that the functional connectivity between left Basal Forebrain (Ch1-3) and the left postcentral gyrus was positively correlated with AVLT_N5 scores (*r* = 0.255, *p* = 0.040). The functional connectivity between left Basal Forebrain (Ch4) and the opercular part of the left inferior frontal gyrus was negatively correlated with BNT scores (*r* = −0.281, *p* = 0.023). Additionally, the functional connectivity between right Basal Forebrain (Ch4) and the left calcarine sulcus was correlated with STT-B scores (*r* = 0.258, *p* = 0.038) (Figure 6).

## 4 Discussion

The basal forebrain has been recognized as a key component of memory-related neural circuitry and has been proposed as one of the three major memory-related circuits, together with the medial temporal lobe system and the Papez circuit (11). In this study, we investigated the functional connectivity of basal forebrain circuits in aMCI patients. Overall, we observed a decrease in functional connectivity across the network. Affected nodes included the bilateral basal forebrain, left sensorimotor cortex, opercular part of the left inferior frontal gyrus, left parietal lobule, left visual cortex, left precuneus, and left supramarginal gyrus. Interestingly, we also found increased functional connectivity between the right basal forebrain Ch4 and several cortical regions.

Left Basal Forebrain (Ch1-3) is composed of MS and DB. MS and ventral diagonal band of Broca (vDB) located in the basal forebrain are anatomically and functionally related to the hippocampus (28). MS is a basal forebrain region that shares bidirectional neuronal connections with the hippocampus and is involved in the neural circuit mechanisms underlying social memory (29). Its degeneration is associated with neurodegenerative diseases and aging (30). The precentral gyrus is traditionally associated with motor functions, while the postcentral gyrus is linked to sensory processing. However, research has shown that the precentral gyrus also plays a crucial role in higher cognitive processes, including attention, learning, and consolidation (31). Additionally, rs-MRI studies have found that voxel-mirrored homotopic connectivity (VMHC) abnormalities in the postcentral gyrus may be mechanistically involved in the pathophysiological progression of patients with MCI (32). This is consistent with our findings, which show reduced functional connectivity between left Basal Forebrain (Ch1-3) and both the precentral and postcentral gyri. This may be related to memory decline in aMCI patients, as both the basal forebrain and the precentral and postcentral gyri are involved in this process.

We also observed reduced functional connectivity between left Basal Forebrain (Ch1-3) and regions associated with the visual network. The visual network plays a critical role in visual signal processing, visual memory, visual learning, and visual-motor coordination. The opercular part of the left inferior frontal gyrus, a subregion of the left frontal lobe, has recently been implicated in audiovisual learning, extending its known role beyond traditional language processing functions (33, 34). Additionally, the calcarine cortex, a core component of the visual network, and the occipital lobe more broadly, are both critically involved in visual information processing. In patients with aMCI, structural and functional changes have been observed in the parietal lobe and middle occipital gyrus, which may reflect early neurodegenerative processes (35). Our findings are consistent with previous studies showing that the occipital lobe is affected in individuals with subjective cognitive decline, and these alterations have been associated with reduced visuospatial ability and executive dysfunction. Furthermore, a recent meta-analysis of brain networks in MCI patients supports our results, reporting reduced activation within the visual network compared to healthy controls (36).

Left Basal Forebrain (Ch4) belongs to the NBM, the primary source of cholinergic innervation to the cerebral cortex (37). It plays an indispensable role in the subcortical regulation of memory, attention, and arousal. Research indicates that with aging, the functional connectivity between the NBM and the visual and somatomotor cortices decreases (38). The precuneus, located in the medial parietal lobe, is a core region of the default mode network (DMN) and is involved in episodic memory retrieval and spatial cognition (39). Studies have found a correlation between the volume of the NBM and glucose metabolism in the right precuneus. A lower volume of the NBM is associated with reduced glucose consumption in the right precuneus (40). Our study found reduced functional connectivity between the NBM and the precuneus, consistent with these findings.

The left supramarginal gyrus, located in the parietal lobe, is part of the ventral attention network and is involved in episodic memory encoding. A resting-state magnetoencephalography study noted that reduced beta-band activity in the left supramarginal gyrus reflects cognitive decline (41). The inferior parietal lobe is associated with spatial attention and semantic memory, participating in higher cognitive functions (42). Our study found reduced functional connectivity between right Basal Forebrain (Ch1-3) and brain regions related to learning, attention, and memory, which may be linked to memory decline in aMCI patients.

Surprisingly, we found that the aMCI group exhibited higher functional connectivity between right Basal Forebrain (Ch4) and the left superior occipital gyrus, left middle occipital gyrus, left precuneus, and left superior parietal lobule compared to the HC group. Functional connectivity between the right basal forebrain Ch4 and the left calcarine sulcus was positively correlated with STT-B scores. Because higher STT-B scores indicate longer completion time and poorer executive/visuospatial performance, this association does not support a beneficial compensatory effect. Instead, it suggests that increased basal forebrain–visual network connectivity may reflect inefficient or maladaptive neural reorganization. Previous studies have shown that increased functional connectivity or hyperactivation in MCI and early Alzheimer’s disease does not necessarily represent successful compensation. Hyperactivation in MCI has been proposed as an early functional signature of Alzheimer’s disease, and individuals showing higher activation may exhibit more rapid subsequent cognitive decline. Increased connectivity in late MCI and Alzheimer’s disease has also been interpreted as a maladaptive short-term mechanism rather than an efficient compensatory process (10, 43). Therefore, the increased right basal forebrain Ch4 connectivity observed in the present study may indicate an unsuccessful attempt to maintain cognitive performance, or a pathological hyperconnectivity pattern related to disrupted network efficiency. This interpretation is consistent with the coexistence of network disconnection and hyperconnectivity during the early stages of cognitive impairment.

Functional connectivity between left Basal Forebrain (Ch1-3) and the left postcentral gyrus was positively correlated with AVLT_N5 scores. The AVLT is a commonly used memory assessment, with AVLT_N5 related to long-delay recall. The precentral gyrus also plays a key role in higher cognitive processes. Functional connectivity between left Basal Forebrain (Ch4) and the opercular part of the left inferior frontal gyrus was negatively correlated with BNT scores. The BNT assesses naming and aphasia (22). The opercular part of the left inferior frontal gyrus, a component of the left frontal lobe, is involved in syntactic processing, and recent studies have expanded its role to include audiovisual learning. Functional connectivity between right Basal Forebrain (Ch4) and the left calcarine sulcus was positively correlated with STT-B scores. Our results indicate that the cortical basal forebrain network is related to memory, language function, and executive function.

## 5 Limitation

Several limitations should be noted. First, our aMCI sample included both single-domain and multiple-domain subtypes, and the limited sample size (66 aMCI patients and 58 HCs) precluded subtype-stratified analyses. Second, the spatial resolution was relatively low for the small basal forebrain nuclei, likely introducing partial volume effects; therefore, the subregional findings should be interpreted with caution. In addition, because Ch1-3 was treated as a combined ROI, the present study could not distinguish the potentially different functional contributions of Ch1-2, which project predominantly to the hippocampus, from Ch3, which projects mainly to the olfactory bulb. Third, consistent with previous reports (44–47), sex is known to influence Alzheimer’s disease risk and rs-fMRI connectivity in MCI. Although sex was balanced between our groups and was not controlled as a covariate, our findings should be interpreted with this in mind, and sex stratified analyses are encouraged in future research. Fourth, no direct measures of cholinergic function were included, and the observed connectivity changes should be interpreted as macroscale network alterations rather than direct evidence of cholinergic dysfunction. Fifth, we did not assess amyloid/tau biomarkers or APOE genotype; thus, the aMCI diagnosis relied solely on clinical and neuropsychological criteria. Finally, the exploratory correlation findings did not survive FDR correction and should be interpreted with caution. Future studies with larger samples, higher-resolution imaging, cholinergic-specific measurements, and longitudinal follow-up are warranted.

## 6 Conclusion

We conducted whole-brain functional connectivity analysis using rs-fMRI, with the basal forebrain as the seed region. Our findings revealed alterations in functional connectivity between the basal forebrain and brain regions associated with memory, visual cognition, attention, and related functions. The results suggest an overall disruption of basal forebrain-associated functional connectivity in patients with aMCI. Increased connectivity involving the right basal forebrain Ch4 may reflect inefficient or maladaptive network reorganization rather than definitive beneficial compensation. These findings indicate that both hypoconnectivity and hyperconnectivity coexist within basal forebrain-related networks in aMCI.

## Data Availability

The raw data supporting the conclusions of this article will be made available by the authors, without undue reservation.

## Footnotes

1
https://www.fz-juelich.de/en/inm/inm-7/resources/tools/jubrain-anatomy-toolbox
